# The Silent Presentation of a Non-functioning Pancreatic Neuroendocrine Tumor: A Case Report

**DOI:** 10.7759/cureus.89256

**Published:** 2025-08-02

**Authors:** Logan Bassoff, Kenneth Kersey, Ian Debus, Daniel Glotzer

**Affiliations:** 1 Clinical Sciences, Florida State University College of Medicine, Tallahassee, USA; 2 Medicine, Florida State University College of Medicine, Tallahassee, USA; 3 Surgery, Cleveland Clinic Indian River Hospital, Vero Beach, USA

**Keywords:** ct enterography, distal pancreatectomy(dp), pancreatic cyst management, pancreatic neuroendocrine tumors, surgery general

## Abstract

Pancreatic neuroendocrine tumors (PNETs) are rare pancreatic neoplasms that can be classified as functioning or non-functioning. Non-functioning PNETs are often asymptomatic and detected incidentally. We discuss the case of a 54-year-old female with a past medical history significant for Crohn’s disease (CD) who underwent a CT enterography in 2022 for follow-up imaging. At that time, a lesion at the pancreatic tail was discovered, and active surveillance was chosen. Two years later, an ER visit for symptomatic cholelithiasis indicated growth of this lesion via MRI and CT. A subsequent biopsy confirmed a well-differentiated grade 1 non-functioning PNET and accompanying pancreatic cyst. Surgical options were discussed, and the patient underwent distal pancreatectomy with splenectomy, and the tumor was successfully excised. The patient had an uneventful post-surgical course.

Non-functioning PNETs are challenging to diagnose due to their subtle symptoms and are often detected at later stages. Imaging modalities like MRI, CT, and endoscopic ultrasound are key in diagnosis. Surgical resection remains the most definitive treatment modality. This report highlights the diagnostic complexity and the potential for varied presentations of non-functioning PNETs, underscoring the need for further research on their characteristics, management, and associations with other pancreatic lesions.

## Introduction

Pancreatic neuroendocrine tumors (PNETs) are uncommon neuroendocrine tumors that can be generally described as functioning or non-functioning. Functioning PNETs are characterized as insulinoma, VIPoma, and gastrinoma and are identified early in life based on increased hormonal secretion [1]. Non-functioning PNETs are classically characterized as either low- or non-hormonal-secreting tumors, typically identified in the fourth-fifth decade of life, and older age at the time of diagnosis often leads to a significantly worse prognosis [1,2]. These are often detected as incidental findings on imaging or become symptomatic due to tumor mass effects. Despite its late diagnosis, the five-year survival rate for all PNET variants has risen from 37% in 2000 to 58.9% in 2013 [2]. These tumors vary based on the location (head of the pancreas vs the tail) and the malignancy potential. The cytologic exam of the biopsied tumors enables their staging and follow-up treatment, if required. PNETs account for 2-10% of all pancreatic neoplasms [3,4]. Non-functioning PNETs have been described in several studies, each detailing a different patient presentation. This highlights the unique nature of these tumors and the uncertainty in identifying them before they cause symptoms or health issues.

## Case presentation

**Figure 1:**
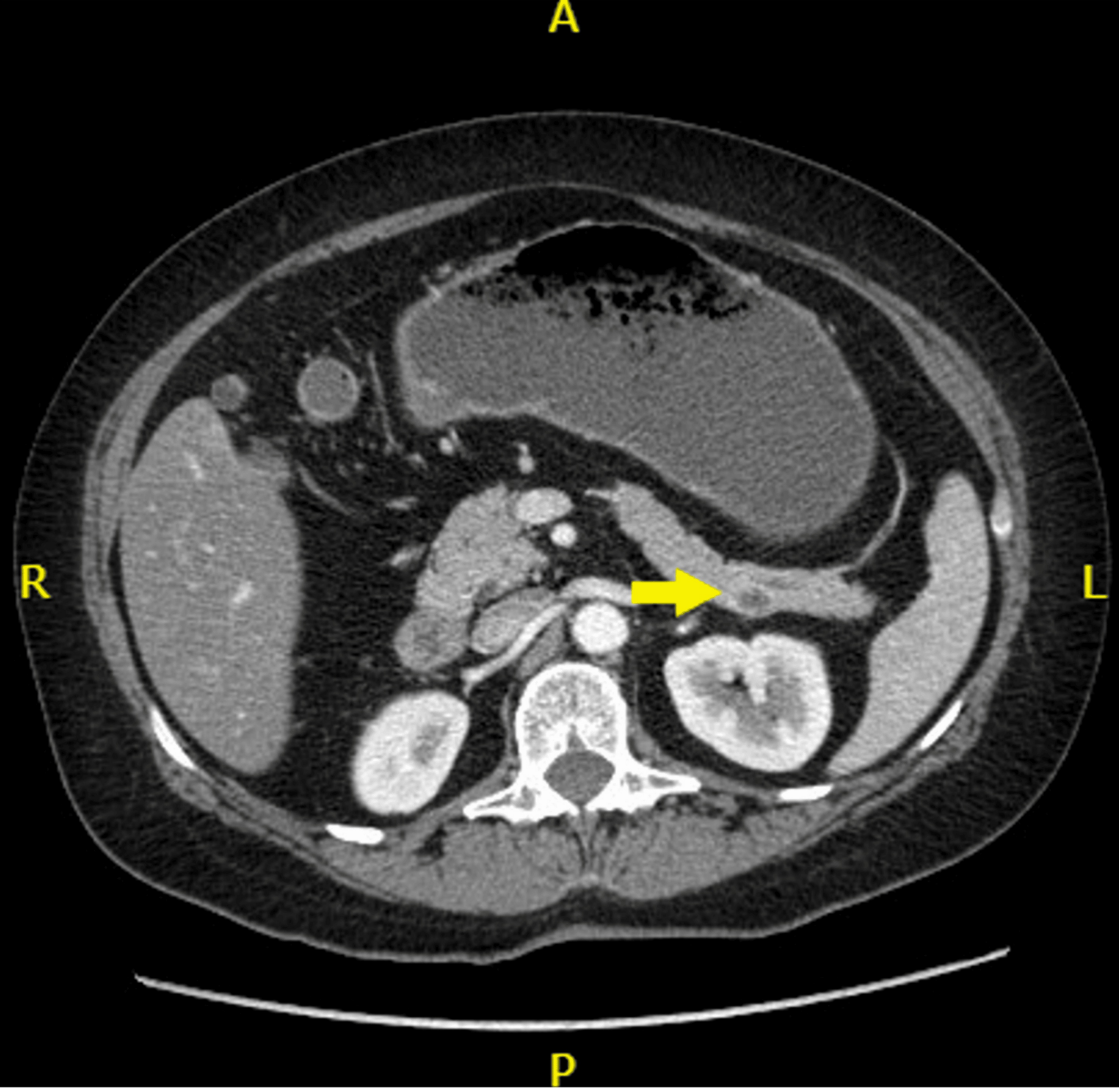
Abdominal CT performed in 2022 CT scan showing 9 x 5 x 5mm solid mass in the tail of the pancreas (arrow). Given the solid, cystic, well-encapsulated nature of the mass, clinical suspicion for malignancy was low. Additionally, the lack of involvement with nearby structures to include the pancreatic duct, and the patient's asymptomatic presentation were accounted for in the decision-making process to reevaluate the mass with close follow-up CT: computed tomography

**Figure 2:**
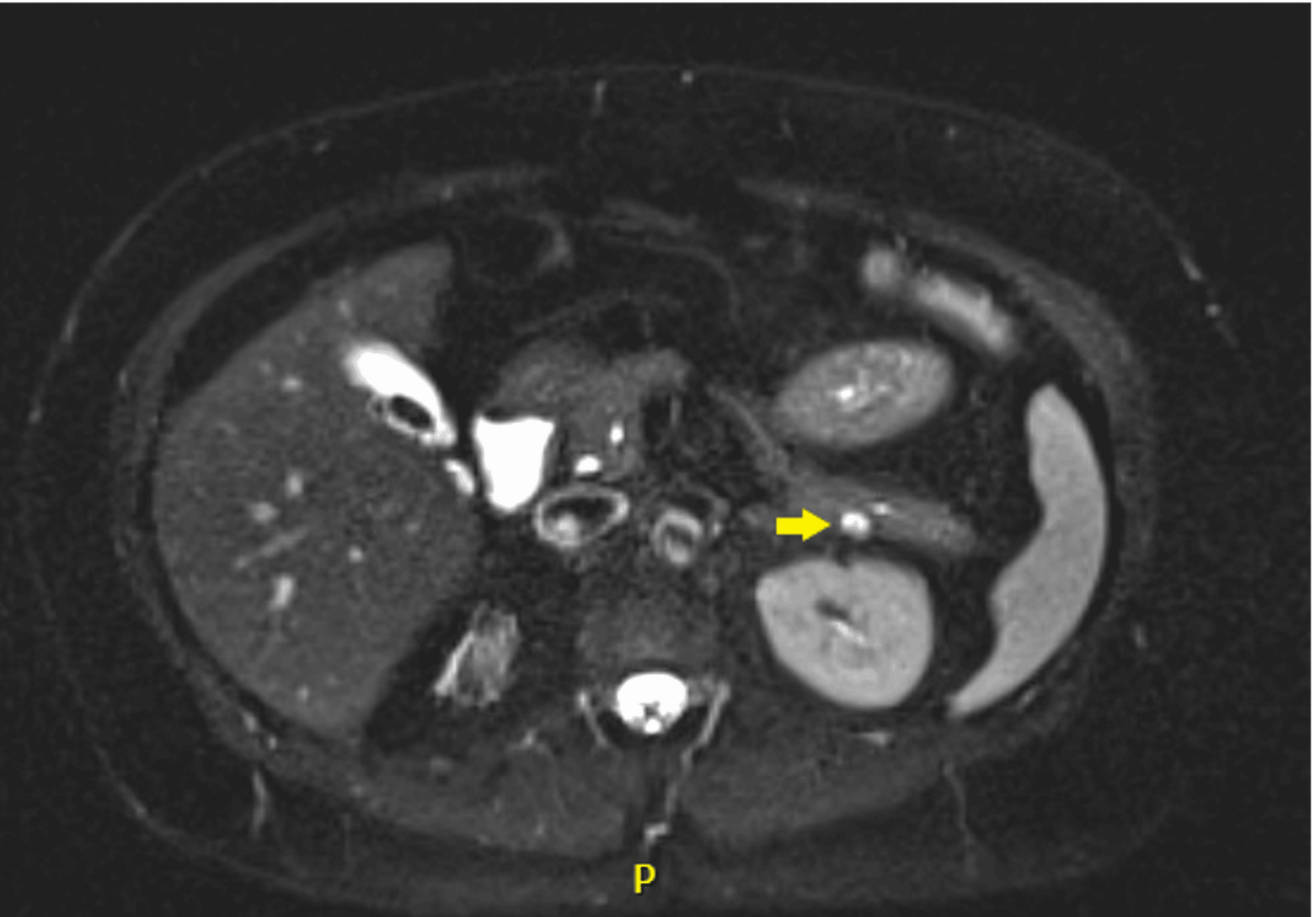
MRI performed in 2024 MRI of the pancreatic mass in 2024 showed increased growth at 13 x 10 mm (arrow). In comparison to the patient's previous CT scan in 2022, the increased growth and presentation of symptoms raised suspicion for a possible malignant lesion MRI: magnetic resonance imaging

The patient was a 54-year-old female with a past medical history of Crohn’s disease (CD), diabetes mellitus type II, and iron deficiency anemia. In 2022, she underwent a CT enterography for evaluation of her CD, which incidentally revealed a singular 9 x 5 x 5 mm hypoechoic, cystic lesion on the posterior pancreatic tail (Figure 1). Given the sub-centimeter size, location of the mass, and the lack of involvement of the pancreatic duct, the clinical suspicion for malignancy was low, and the patient elected for observation and reevaluation with MRI at a later date. Two years later, she presented to the ER with upper abdominal pain, which was subsequently diagnosed as cholelithiasis. An upper endoscopic ultrasound (EUS) confirmed the stones in the gallbladder and allowed the pancreatic mass to be biopsied. An MRI at the time of her ER visit identified a solid cystic mass measuring 13 x 10 mm in maximal cross-sectional diameter, indicating advancement of her disease (Figure 2). The histopathologic findings of the specimen were positive for a well-differentiated neuroendocrine tumor, staining positive for CK AE1/AE3, chromogranin, and synaptophysin.

Treatment options were discussed with the patient, which were surgery or continued observation. Surgery was performed the same day with complete excision of the mass via a distal pancreas, spleen, and omentum resection. Pathology of the mass revealed a Grade 1 non-functional PNET with <3% Ki67. Additionally, a large benign pancreatic cystic lesion was discovered and was described as having a fibrocystic wall, hemorrhage, and granulation tissue. Lymph node and omentum testing identified no metastasis of the tumor. A cholecystectomy was simultaneously performed with no malignancy noted. A follow-up one week later demonstrated the complete resolution of symptoms with minimal residual pain.

## Discussion

Non-functioning PNETs are more difficult to diagnose due to their lack of symptoms and are usually detected incidentally during imaging for other purposes, as seen in our case. Their symptoms also depend on the location of the PNET. PNETs are more often located in the head of the pancreas and cause symptoms such as jaundice and weight loss [3]. The diagnosis involves various imaging modalities such as CT, MRI, EUS, and somatostatin receptor scintigraphy (SRS). Diagnosis with an MRI can be more useful in detecting liver metastasis when compared to CT or SRS [4]. This is noteworthy, as non-functioning PNETs are detected at more advanced stages when the tumor has metastasized or grown large enough to cause mass effect [4]. Testing of the biopsied mass is important to determine if the tumor has subclinical hormonal secretion and would require further follow-up. Common PNET markers are synaptophysin, chromogranin A, neuron-specific enolase, and pancreastatin [1,4]. Our patient's PNET was biopsied at the time of EUS and confirmed via histopathology. In cases where PNET suspicion is high and a pancreatic mass is present, the evaluation of PNET markers is crucial for early identification and diagnosis.

The treatment of a PNET is determined in part by its location and stage at the time of diagnosis. Multiple organizations, such as the European Neuroendocrine Tumor Society (ENETS) and American Joint Committee on Cancer (AJCC), define the staging of these tumors. These agencies have developed a grading system for PNETs [4]. These systems follow traditional TNM-based staging, identifying the primary tumor, lymph node status, and distant metastasis, which play a vital role in the individualized treatment plan of patients. The treatment options for a PNET range from active surveillance to surgery +/- medications. Previously, a surveillance approach was deemed appropriate in cases of non-functioning, asymptomatic PNETs measuring less than 2 cm [5]. However, there is a lack of guideline-related consensus for this intervention. Assessment of tumor size, location, functionality, and patient status is key for determining treatment options [6]. To date, surgery is the only curative treatment for these tumors. Surgical intervention differs depending on the location of the tumor. Historically, tumors in the head of the pancreas have undergone a pancreatoduodenectomy, while a tumor in the tail requires a partial pancreatectomy and splenectomy [3]. Our patient was given the option to have surgery or complete follow-up surveillance based on the diagnosis of a grade 1 non-functional PNET.

There is no available data to explain the sporadic nature of non-functioning PNETs, but up to 10% of PNETs are associated with inherited genetic syndromes [3]. PNETS are a common finding in individuals with hereditary conditions such as multiple endocrine neoplasia syndrome type 1 (MEN1), von Hippel-Lindau disease (VHL), neurofibromatosis type 1 (NF1), and tuberous sclerosis (TSC) [7]. In these instances, the etiology and pathogenesis of a PNET are better understood compared to non-functioning PNETs, given their association with related hormone secretion (functional PNETs).

Previous epidemiologic studies have shown a higher incidence of functional PNETs compared to the non-functional variant [8]. When compared between sexes, the overall incidence was 1.8 per million in females and 2.6 per million in males [8]. Non-functioning PNETs have also been found to be most prevalent during the eighth decade of life in both males and females [8]. Unlike functional PNETs, the survival rate in patients with non-functioning PNETs is substantially lower. Non-functioning PNETs have a 5- and 10-year survival rate of 33.7% and 17%, respectively [8], in contrast with the 5- and 10-year survival rate of functioning PNETs at 41.6% and 31.3%, respectively. This further emphasizes the need for earlier identification and surgical management of these tumors [8]. It is known that CD and ulcerative colitis (UC) can cause extra-intestinal problems with the joints, skin, and eyes, among other systems. This multisystem involvement can lead to increased rates of pancreatitis, pancreatic duct abnormalities, and antibodies against pancreatic tissue in those with CD [9]. Hedfi et al. have described a case of a male with longstanding CD who was found to have a neuroendocrine tumor of the tail of the pancreas, suggesting a possible link between CD and PNETs [10].

Another unique finding in our patient was the presence of a large, benign pancreatic cystic lesion that was discovered after the surgical removal of the tumor. A preliminary literature search identified only one case report that discusses a simultaneous presentation of a PNET and a pancreatic cyst. The presence of the cyst and its local inflammatory reaction changed the operative plan: from attempting a spleen-preserving distal pancreatectomy to an en bloc resection of the distal pancreas, including the tumor and the spleen. Our patient underwent a distal pancreatectomy and splenectomy. The final pathology showed a pancreatic simple mucinous cyst with a grade 1 PNET infiltrating the cyst wall [11]. We highlight the scarcity of literature on the varying presentations of PNETs in the tail of the pancreas, which can be easily misidentified.

## Conclusions

PNETs pose significant diagnostic challenges, particularly when they are non-functioning, as their lack of hormonal secretion means that they are often incidentally detected during unrelated imaging. This report demonstrates the complexity of diagnosing non-functioning PNETs, mainly due to their silent clinical presentation and incidental identification. The concomitant finding of a pancreatic cyst in our patient added another layer of diagnostic complexity. Surgical intervention remains the primary curative treatment for these tumors, as demonstrated in this case, where a distal pancreatectomy and splenectomy were performed. The concurrent discovery of a benign pancreatic cyst further highlights the variability in PNET presentations and the need for more research on their characteristics and management strategies, particularly regarding non-functioning PNETs. This report emphasizes that clinicians should maintain a high index of suspicion for tumors, especially silent tumors, when incidental masses are found in the pancreas. Although a cystic mass may be asymptomatic, clinicians should still investigate and evaluate for signs of functioning or non-functioning PNETs in their patients.
